# Protective Factors against Self-Harm and Suicidality among Australian Indigenous Adolescents: A Strengths-Based Analysis of the Longitudinal Study of Indigenous Children

**DOI:** 10.3390/ijerph19159131

**Published:** 2022-07-26

**Authors:** Md Irteja Islam, Lisa Sharwood, Verity Chadwick, Tuguy Esgin, Alexandra Martiniuk

**Affiliations:** 1 Sydney School of Public Health, Faculty of Medicine and Health, The University of Sydney, Edward Ford Building, A27 Fisher Road, Sydney, NSW 2006, Australia; alexandra.martiniuk@sydney.edu.au; 2 Centre for Health Research, and Faculty of Health, Engineering and Sciences, The University of Southern Queensland, West Street, Darling Heights, Toowoomba, QLD 4350, Australia; 3 Faculty of Medicine and Health, The University of Sydney, Sydney, NSW 2006, Australia; lisa.sharwood@sydney.edu.au; 4 Faculty of Medicine and Health, University of New South Wales, Sydney, NSW 2032, Australia; 5 Faculty of Engineering and Information Technology, University of Technology Sydney, Sydney, NSW 2007, Australia; 6 Royal North Shore Hospital, Reserve Rd., St. Leonards, Sydney, NSW 2065, Australia; veritychadwickjackman@gmail.com; 7 Discipline of Exercise and Sports Science, Faculty of Medicine and Health, The University of Sydney, Level 6 Susan Wakil Health Building D18, Western Ave, Camperdown, Sydney, NSW 2050, Australia; tuguy.esgin@sydney.edu.au; 8 School of Medical and Health Sciences, Edith Cowan University, 270 Joondalup Drive, Joondalup, WA 6027, Australia; 9 School of Management and Governance, and UNSW Business School, University of New South Wales, Kensington, Sydney, NSW 2052, Australia; 10 Office of the Chief Scientist, The George Institute for Global Health, Level 5/1 King Street, Newtown, Sydney, NSW 2042, Australia; 11 Dalla Lana School of Public Health, The University of Toronto, 155 College St. Room 500, Toronto, ON M5T 3M7, Canada

**Keywords:** indigenous peoples, adolescent, adolescent health, self-injurious behaviour, suicide, health and wellbeing

## Abstract

**Background:** Understanding and encouraging social and emotional well-being (SEWB) among Indigenous adolescents is vital in countering the impacts of colonisation and intergenerational trauma. As self-harm and suicidality are considered markers of poor SEWB among Indigenous communities, we aimed to identify the individual-level and community-level factors protecting Indigenous adolescents from self-harm and suicidality. **Methods:** Data came from Footprints in Time—The Longitudinal Study of Indigenous Children (waves 10 and 11), conducted among Indigenous families across Australia. A strengths-based analysis fitted multilevel logistic regression to explore associations with factors proposed as protective against self-reported self-harm and suicidality among Indigenous adolescents. **Results:** Our study cohort included 365 adolescents with complete data for the variables of interest. Adolescents had a mean (SD) age of 14.04 (0.45) years and a sex ratio of almost 1:1, and most were attending school (96.2%). Previous self-harm was reported by 8.2% (*n* = 30); previous suicidality was reported by 4.1% (*n* = 15). Individual-level factors protecting against self-harm and suicidality were being male, living in a cohesive family, and having low total Strengths and Difficulty Questionnaire scores (*p* < 0.05 for all). Residing in major cities compared with regional/remote areas was protective against self-harm (OR 5.94, 95% CI 1.31–26.81). Strong cultural identity was not found to be a protective factor against self-harm and/or suicidality in the sample. **Conclusions:** This study identified key individual- and community-level factors that can protect Australian Indigenous adolescents against self-harm and suicidality, particularly family cohesion. Identifying strengths for this at-risk population can inform prevention strategies, particularly for rural living adolescents with high distress.

## 1. Introduction

Suicide and self-harm represent serious global health problems and are notably elevated amongst Aboriginal and Torres Strait Islander people (hereby respectfully referred to as Indigenous Australians). Indigenous Australians are one of the oldest living cultures in the world, but this once-healthy culture has been adversely affected by colonisation and intergenerational trauma. The adverse effects of colonisation are many and include self-harm and suicidal ideation as markers of considerable emotional distress. This research responds to a priority determined by Indigenous communities. Suicide rates among Indigenous Australians are up to four times higher than their non-Indigenous counterparts [1]. In some remote communities in the Kimberley, rates of suicide have reached one hundred times the national suicide average [2]. Risk factors include incarceration, substance use and the experience of social and emotional distress. Information on protective factors against suicide and self-harm is scarce yet much needed to encourage communities in prevention efforts. There is a gradual move towards research empowerment within and by Indigenous communities as a means of healing, encouraging both acceptance of past harms and motivation to strengthen community capacity [3].

Prior to European colonisation in Australia, raising children was a shared responsibility within communities, and each child was treasured by the group as special, knowing the pride of being Aboriginal, being black, experiencing and learning the cultural values of work, safety, food and shelter, honour, and truthfulness [4]. The impacts of colonisation have been heavily published [5,6,7,8]; importantly, colonisation has resulted in the separation and fracture of Indigenous communities, changing this practice of child-raising indefinitely [6,7]. It is recognised that a general impact of colonisation is the predominantly negative discourse and focus on blaming the Indigenous culture for the gaps in healthcare outcomes [9]. There is, however, a consensus that the impacts of colonisation on social justice for Indigenous people are overly complex and have affected their health intergenerationally [10,11]. Estimating inequalities can provide important baselines from which to measure change [12] but often insufficiently counts population outcomes as a single measure, masking the diversity within populations, and revealing little about how to sustainably improve health and wellbeing.

Studies have shown that connectedness to land, culture, and language is protective in terms of health including mental health or, as Indigenous people prefer, Social and Emotional Well-Being (SEWB) [13]. Connectedness to healthy land is essential for Indigenous peoples’ health and well-being. Connectedness is a feeling and may be fostered through activities such as time on country, grass burning, gathering food and medicines, ceremony, and protecting sacred areas and artwork. New community-driven programs that incorporate this importance of culture have been shown to be effective in reducing suicidality in Indigenous youth and provide hope for fostering stronger SEWB in future [14].

There are many great initiatives working to improve SEWB in Indigenous communities, including community-led Aboriginal and Torres Strait Islander Mental Health First Aid courses, language programs, and Indigenous suicide awareness and prevention programs [15,16]. Despite such efforts, evidence suggests a continued higher risk of emotional and/or behavioural problems among Aboriginal adolescents, around four times higher than the risk among non-Aboriginal adolescents [17], and suicide rates are almost 13 times higher among Indigenous adolescents than non-Indigenous adolescents [18]. These facts contributed to the prioritisation of suicide prevention for Indigenous young people in the National Aboriginal and Torres Strait Islander Suicide Prevention Strategy [19]. Previous research found that risk factors for self-harm and suicide among Indigenous adolescents include living outside of the parental home, living in remote or very remote areas, consuming alcohol, and not receiving treatment for psychiatric disorders [20]. However, much of this past research has focused on the deficits rather than the strengths of Indigenous communities and how these strengths can be employed to mitigate suicide and self-harm. Possessing a strong cultural identity has been shown to protect against mental health symptoms and buffer distress prompted by discrimination [21]. It has been previously hypothesised that a strong cultural identity might protect against self-harm and suicidality, as this has been shown to be true for other First Nations populations [21,22]. More research examining protective factors in Australian Indigenous communities as well as in other cultures has been called for [23]

This research meets an urgent need determined by Indigenous communities; the aim of this study was to identify factors that may protect Indigenous adolescents from self-harm and suicidality using a strengths-based approach.

## 2. Methods

### 2.1. Data Source

Footprints in Time: The Longitudinal Study of Indigenous Children (LSIC), is an ongoing national prospective cohort study funded and managed by the Australian Government Department of Social Services [24]. The overall objective of the LSIC study is to offer greater insight into the lives of Indigenous children in Australia, aiming to inform efforts to close the gap in life and health outcomes between Indigenous and non-Indigenous Australians with specific aims articulating the search for strengths in Indigenous children and adolescents as they grow. Key research questions underlying the LSIC data collection are “What do Indigenous children and adolescents need to have the best start in life to grow up strong?” and “What helps Indigenous children and adolescents to stay on track or get them to become healthier, more positive, and strong?” [8]. It is from this perspective that we applied a strengths-based approach, previously described by [9], in the current study.

**Figure 1:**
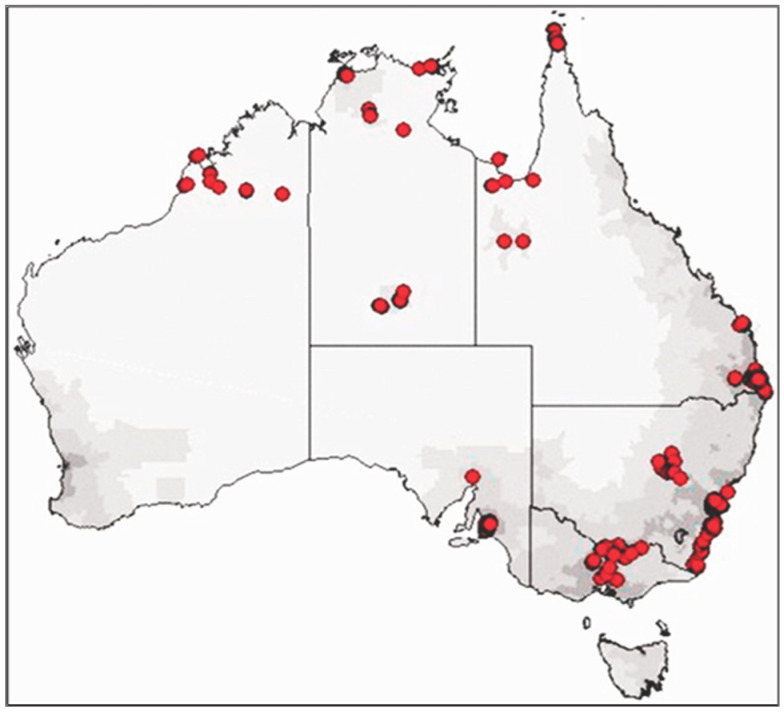
LSIC sample distribution.

The study design used for the LSIC has been previously described [24,25,26]. However, briefly, LSIC used a non-random purposive sampling design across 11 Indigenous communities in Australia, following longitudinally the growth, development, and specific outcome measures of 1700 Indigenous families (parents, carers, and adolescents) in urban, regional, and remote settings. Figure 1 shows the location of participating families in the LSIC.

The first wave of the interviews and survey commenced in 2008 with two groups of Indigenous children—the younger B-cohort (aged 0–1.5 years at baseline) and the older K-cohort aged (3.5–5 years at baseline)—and these children participated in subsequent waves conducted annually. Data were collected through face-to-face interviews between an Indigenous interviewer and the participants (i.e., the study child, parent, or teacher). 

### 2.2. Participants 

**Figure 2:**
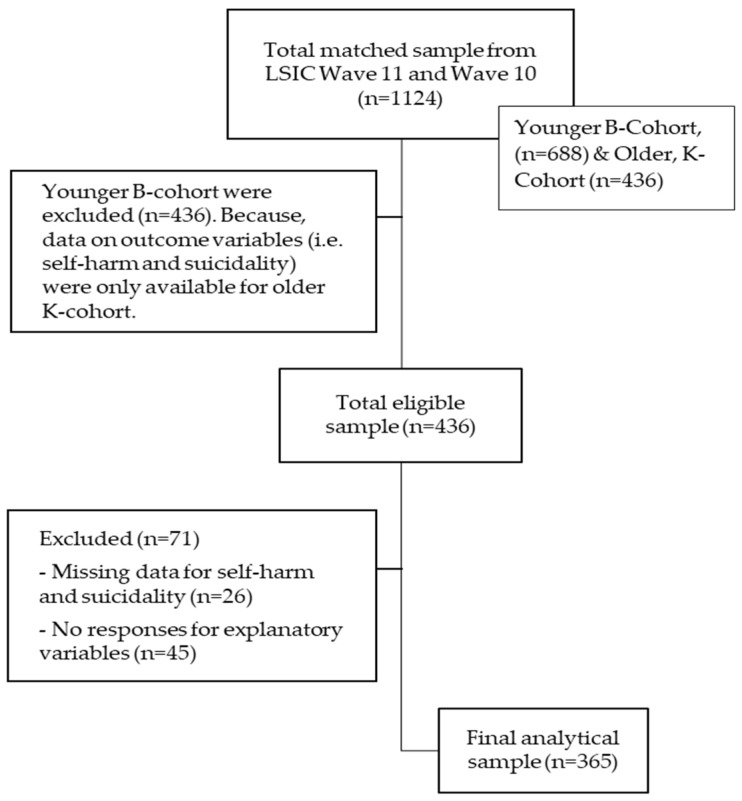
Flow chart for sample selection.

A flow chart for the selection of the analytical sample is presented in Figure 2. The current study included 365 Indigenous adolescents aged 13.5–15 years at the time of the latest LSIC Wave 11 in 2018. We included participants who provided complete data on the outcome variable (i.e., self-harm and suicidality) and exposure variables in our study. Participants who did not respond to the outcome or predictor variables were omitted (*n* = 71).

### 2.3. Measures

A range of sociodemographic variables as well as variables associated with social and emotional well-being were examined in relation to self-harm and suicidality among Indigenous adolescents aged 13.5–15 years using the ‘Positive Outcome Approach’ [9]. For example, this approach measures the association between protective factors (e.g., employed parents, or resilience) and positive outcome variables (e.g., strong social and emotional well-being) instead of using risk factors (e.g., unemployed parents or high alcohol use) and adverse outcome variables (e.g., poor mental health) [9]. 

The variables included in this study are listed in Table 1. 

### 2.4. Cultural Integrity 

This research provided the opportunity for Indigenous and non-Indigenous authors to learn from each other. It provided the Noongar/Yamatji Aboriginal co-author (TE) with the opportunity to build his research capacity and benefit from his leadership, experience, and knowledge regarding Indigenous knowledge. It also provided the opportunity to govern, share, maintain, and grow his cultural and intellectual heritage using Indigenous ways of knowing, being, and doing in guiding the research processes. Although the research did not use an Indigenous research paradigm due to its quantitative nature, it was influenced using a strengths-based model and incorporated aspects of the CREATE Aboriginal and Torres Strait Islander quality appraisal tool [38] to comply with aspects of cultural integrity where possible. 

### 2.5. Statistical Analysis

Sample descriptive statistics were calculated using Pearson’s Chi-square test of independence to investigate bivariate associations between each explanatory variable and outcome variables. For the multivariable analysis, considering that the study sample (i.e., Indigenous children) was nested in households and households were nested within clusters in LSIC [25], we used multilevel mixed-effects logistic regression models to examine the associations of potential protective factors with no reporting of self-harm and suicidality, respectively.

We fitted four models to estimate both fixed effects of the individual and community level variables and a random effect (cluster) for the unexplained variability between clusters. Model 1 was the empty or null model (without explanatory variables) to assess random variance in the intercept. Then, all individual-level variables were included in Model 2, and community-level variables were added to Model 3. Finally, Model 4 was fitted for both individual and community level variables simultaneously. Variables yielding a *p*-value of <0.05 in the bivariate analysis were included in the multivariable analysis. The measures of cluster variation were estimated as the intraclass correlation coefficient (ICC); an ICC ≥ 2% was considered as a minimum precondition to conduct the multilevel modelling [39]. Further, we computed the median odds ratio (MOR) and proportional change in variance (PCV) to quantify unexplained cluster variability [40]. We also estimated the Bonferroni-adjusted *p*-values for multiple comparisons for the multivariate analysis as a conservative comparison of significance [41]. Lastly, we used Akaike’s information criterion (AIC) to assess the goodness of fit of each model and the variance inflation factor (VIF) to check multicollinearity between explanatory variables. Stata/SE 14.1 (Stata Corporation, College Station, TX, USA) was used to perform all statistical analyses, and statistical significance was set to 0.05.

## 3. Results

### 3.1. Sample Characteristics 

Our study cohort included 365 adolescents with complete data for the outcome and predictor variables of interest, with a sex ratio of almost 1:1. Adolescents had a mean (SD) age of 14.04 (0.45) years, and most reported attending school (96.2%). Most respondents reported having good family cohesion (80.8%), 69.6% of adolescents thought that Indigenous identity is important, and the majority of the sample participants were able to make friends easily (88.8%). Nearly 90% of adolescents reported high self-efficacy and more than three-quarters (77.8% had average/high SDQ prosocial subscale scores. More than two-thirds of the adolescents came from regional/remote areas (68.5%), and almost 42% of the sample (combining Q4 and Q5 of IRSEO index) were socioeconomically advantaged. The characteristics of our study population are shown in Table 2.

### 3.2. Bivariate Analyses

**Figure 3:**
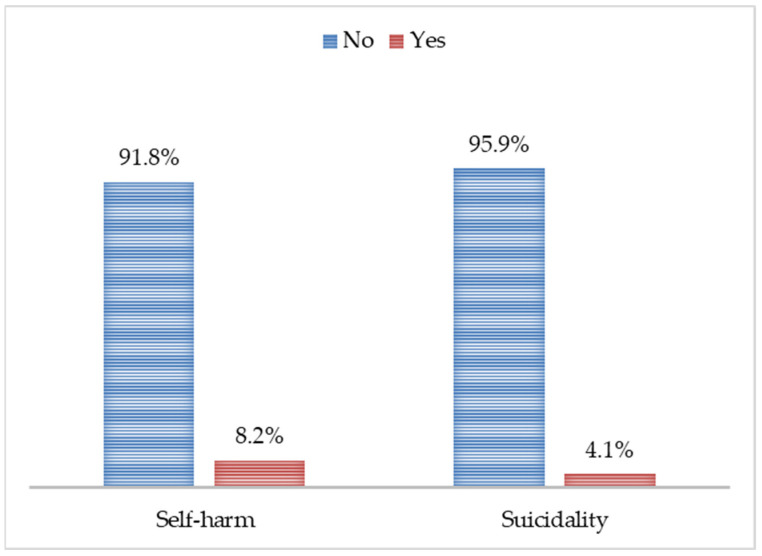
Proportion of self-harm and suicidality (yes vs. no).

The proportion of adolescents who did not report a history of intentional self-harm was 91.8%, and the proportion of adolescents who did not report previous suicidality was 95.9%, shown for the study sample in Figure 3. 

The findings from the bivariate associations of potential protective factors against self-harm are portrayed in Table 3. Among the individual-level factors, the sex of the study child (no self-harm reported by 53.5% of boys vs. 46.65 of girls), ‘strong’ family cohesion, making friends easily, and average/high SDQ pro-social subscale scores were significantly protective against self-harm (*p* < 0.05 for all). Among the community-level factors, regional/remote area of residence was more likely to be protective against self-harm (*p* = 0.008). 

The findings from the bivariate associations of potential protective factors against suicidality are portrayed in Table 4. Among the individual-level factors, the sex of the study child (no suicidality reported by 52.3% of boys vs. 47.7% of girls), ‘strong’ family cohesion and making friends easily were significantly protective of suicidality (*p* < 0.05 for all). Among the community level factors, regional/remote area of residence was more likely to be protective against suicidality. Seeing Indigenous identity as important and self-efficacy were not found to be associated with self-harm or suicidality among Indigenous adolescents aged 13.5–15 years.

### 3.3. Multilevel Analyses

The fixed effects (measure of association) and the random intercepts for self-harm are presented in Table 5. Model 1 (the empty model) revealed that clustering existed in determining self-harm. The ICC of the empty model indicated about 38.4% of the total variance in the outcome could be attributed to differences between clusters. Table 5 also indicated the presence of unexplained cluster heterogenicity, considering the values of the MOR and PCV. For example, the unexplained cluster variation in self-harm was decreased to an MOR of 2.94 when all variables were added to the empty model in Model 4. Moreover, as depicted by the PCV, 48.1% of the variance in self-harm across clusters was explained by the individual-level factors (Model 2), and 17.3% of the variance in the outcome was attributable to the community-level factors (Model 3). Multilevel analysis (Model 4) showed that boys were 3.95 times (95% CI: 1.44, 10.81) less likely to report self-harm compared with girls. Strong family cohesion was found to be protective against self-harm compared to their counterparts (OR 4.41, 95% CI: 1.69, 11.44), and average/high SDQ pro-social subscale scores were significantly associated with lower odds of reporting self-harm compared with low/slightly low SDQ pro-social subscale scores (OR 3.30, 95% CI: 1.03, 11.78). In addition, Indigenous adolescents who were living in urban areas were less likely to report self-harm compared with those who were living in regional/remote areas (OR 6.97, 95% CI: 1.34, 36.10). 

In Table 6, the results of the multilevel logistic regression models for suicidality are shown. Table 6 shows the significant variation in the odds of suicidality across clusters and confirmed cluster heterogeneity (ICC 44.2%) and unexplained cluster variability using MOR and PCV estimates. As expected, the multilevel analysis in Table 6 revealed similar findings to that of intentional self-harm, except for the SDQ pro-social subscale score and community-level factor (area of residence).

## 4. Discussion

We found that individual-level factors protective against self-harm and suicidality for Indigenous adolescents included male gender, strong family cohesion, and average/high SDQ pro-social subscale scores (not for suicidality). Only one community-level factor (living in urban areas) was found to be protective against self-harm. Contrary to other research, the importance of Indigenous identity was not a protective factor for self-harm or suicidality. Similar to other studies [42], we found self-harm to be more prevalent than suicidality, reflecting patterns observed among Australian non-Indigenous youth [43,44]. 

It has been well-acknowledged that self-harm and suicidality are more common in female than male teenagers globally and in Australia [44,45,46,47,48]. This may be because anxiety and depression are more prevalent among girls compared with boys, which may lead to self-harming and/or suicidal behaviours [47,49,50], although this trend tends to even out in later teenage years. Though research has shown that males are more likely to commit suicide and women are more likely to attempt [51]. There are likely gender differences in the self-reporting of these behaviours and thoughts as well, though this has been less often studied.

The term family cohesion describes the level of commitment, help, and support each family member provides for one another [52,53]; this study revealed strong family cohesion to be protective against self-harm and suicidality among Indigenous adolescents. This is consistent with findings from other non-Indigenous cohorts [54,55,56]. Evidence suggests that adolescents may learn to manage negative emotions as they gain more family support from cohesive families [53,57]. Additionally, adolescents living in a cohesive family may value family harmony over self-autonomy; this is associated with lower levels of psychological distress, and consequently, this may act as a protective factor against self-harm and suicidality among adolescents [58,59]. 

This study found that Indigenous adolescents with average/high SDQ pro-social subscale scores (i.e., measuring empathy and concern for others) [31] are significantly less likely to report self-harm than those with low/slightly low SDQ pro-social scores. Evidence suggests that children and adolescents with average/high SDQ pro-social scores are more likely to report better social connectedness and relationships (factors related to social and emotional well-being) and fewer mental health problems [31,60,61]. Previous analyses in this study cohort only explored the relationship between suicidal ideation and SDQ prosocial subscale scores, finding no statistically significant associations [31]. Similarly, no clear association between prosocial behaviours and suicidality have been reported in Spanish adolescents [62,63]; our paper extends this finding to Indigenous youth in Australia.

Living in major cities was a protective factor against adolescent self-harm at the community level. Lower suicide rates in metropolitan cities have been attributed to lower socio-economic inequality, greater access to services, and lower rates of unemployment and alcohol consumption [64,65,66,67]. Recently, AIHW 2022 reported that rural Indigenous people are more likely to commit suicide than those from urban areas [68]. Indigenous people have guided Indigenous-specific suicide prevention interventions and have highlighted that the focus of self-harm and suicide prevention programs needs to include the community rather than just focus on the individual child or young person [69]. Indigenous elders in Canada reporting on similarly varied rates of intentional self-harm in some communities and not others suggested this as closely correlated with the strength of connection to community and culture [70]. 

To our surprise, in our study, parents’ perception of how much their children valued cultural identity was not a protective factor against self-harm or suicide. Evidence suggests that cultural identity is a complex and changing concept for adolescents from a life-course perspective and is an integral aspect of development during the transition from adolescence to adulthood [71,72]. Cultural identity is thought to strengthen young people’s self-identity and sense of connectedness with their family and community [73]. Moreover, it has been found that Indigenous people’s cultural identity strengthens resilience, builds self-esteem, and fosters pro-social coping mechanisms, which are essential for mental health and wellbeing [21,68,74]. Berker et al. explored the emerging evidence base for “culture as treatment” to prevent suicide, emphasising the ‘significance of interconnectedness in healing and revitalisation of traditional values to reclaim community wellness’ [75]. However, community can also play a leading role in Indigenous youths arriving at a self-reinforcing cycle of emotional injury and self-harm, particularly in communities where adolescents are not exposed to positive role models and the social persuasions of older generations, instead having similarly disconnected peers and developing a shared collective normalisation of suffering [73]. Indigenous concepts of ‘self’ move beyond dominant western concepts of the individual. Indigenous self-continuity is collective, relational, multidimensional, and connected to strong cultural continuity to the past, present, and future [76]. However, it is this very fabric of Indigenous self-continuity t has been targeted and eroded by colonialism, as a lack of positive affective attachment to the past (nostalgia or pride, for example) appears to foreclose a future—a ‘lack of past-to-present self-continuity is associated with suicidality’—implying an ultimate level of disregard for one’s future self [75,77,78].

These findings are important, as they shed light on protective factors for self-harm and suicidality in Indigenous populations and can provide guidance for future policies targeting this area of need. A person’s health is the result of a complex web of interactions, with biological, social, societal, and environmental factors working together to influence mental health. Knowing people who engage in self-harm or have died by suicide significantly increases one’s own risk of engaging in self-harm or suicide [42,70], and these actions have a perpetuating effect on communities; it is vital to halt the cycle to minimise the ongoing impact. One way to improve rates of self-harm and suicide in Indigenous communities could include community leadership of culturally sensitive educational programs, and efforts to re-engage and promote youth’s cultural connectedness to overcome the impacts of colonisation with a strengths-based lens.

This study was not without its limitations. Self-harm and suicidality were self-reported by the Indigenous adolescents, and therefore, the results may include recall bias and social desirability bias. However, studies have found young people who engage in self-harm/suicidality often do not disclose it to family or friends but may be more likely to report it in writing; therefore, survey data may be a better method to elicit this information compared to directly asking young people verbally [42]. Furthermore, suicidality-related information in the data was limited and could not be distinguished between ideation, plan and/or attempts. As we know that Indigenous people tend to seek healthcare less often than their non-Indigenous counterparts; healthcare utilisation data most likely undercounts self-harm and suicidality in Indigenous youths [79]. Our sample size was small (only 365 participants) and nationally not representative, although participants were recruited from eleven sites across Australia. In addition, there is a possibility of overlap between self-harm and suicidality, which might over/underestimate the results. Both a strength and a limitation of our study is our use of a strengths-based approach to understand the factors protecting Indigenous youth from self-harm and suicidality. We expected to see that identifying cultural identity as important would be protective, but we did not observe this. This finding requires further unpacking. Future work will benefit from learning about and measuring factors associated with good social and emotional well-being within and beyond health variables, going further than simply looking at low scores on measures of pathology.

## 5. Conclusions

A strengths-based approach found strong relationships, with strong family cohesion and being male identified as protective against both self-harm and suicidality. In addition, average/high SDQ pro-social subscale scores and living in the city were protective against self-harm for Indigenous youth. These findings highlight the importance of family connectivity in supporting good social and emotional well-being. A greater understanding of the cultural context of mental health problems such as self-harm and suicidal behaviour in Indigenous youth may help mental health services improve access and remove barriers to treatment, address needs for culturally competent care, and improve the quality of care for Indigenous populations. 

## Figures and Tables

**Table 1:** List of variables.

Variables	Description of Variables
Outcome variables	
Self-harm and Suicidality	Items regarding self-harm and suicidality were directly asked (with consent from parents/caregivers) to adolescents in the K-cohort only in Wave 11 of the LSIC survey. Self-harm was measured by the question: ‘Have you ever deliberately done something to yourself to cause harm or injury, without intending to end your own life?’. The item assessing suicidality was: ‘During the past 12 months, did you ever seriously consider attempting suicide?’. Response options for both self-harm and suicidality were categorised as ‘Yes’ (coded 0) and ‘No’ (coded 1). Note that there is a possibility of overlap between the two populations (self-harm and suicidality).
Explanatory variables ^1^	
Individual-level factors	
Age	Age was used as a continuous variable
Sex	Sex of the adolescents was categorised into ‘Girls’ (coded as 0) and ‘Boys’ (coded as 1).
Schooling	Schooling was categorised into three categories: ‘Not in school’ (coded as 0), ‘Private/Catholic school’ (coded as 1), and ‘Public school’ (coded as 2).
Family cohesion	Family cohesion was measured by the following question—‘Does [study child’s] family get along well with each other?’ In this study, we created a binary variable ‘family cohesion’. Those who responded, ‘very good’ or ‘good’ were classified as ‘’Strong’ (coded as 1), while those who answered ‘fair’ or ‘poor’ were classified as ‘Poor’ (coded as 0).
Indigenous identity	Categorised into ‘Not so important’ (coded as 0) and ‘Important’ (coded as 1). According to the National Strategic Framework of Health for Indigenous Australians, Indigenous identity is one of the vital components of SEWB [27].
Making friends easily	Dichotomised into two categories: ‘No’ (coded as 0) and ‘Yes’ (coded as 1).
Self-efficacy	Categorised into two categories: ‘Low’ (coded as 0) and ‘High’ (coded as 1).
The Strength and Difficulties Questionnaire (SDQ)—Prosocial subscale score	The SDQ questionnaire is a globally used screening tool for social, emotional and behavioural challenges among children and adolescents aged 2–17 years [28,29]; whereas, for example, Parents’ Evaluations of Developmental Status (PEDS) can only be utilised for children aged 0–9 years [30]. The SDQ consists of four difficulty subscales (emotional symptoms, conduct problems, hyperactivity, and peer problems), and one strength subscale (prosocial behaviour) [31]. Since previous studies have found that the SDQ is internally consistent, reliable, and valid among Australian Indigenous children [32,33]; the LSIC used different age-appropriate versions of the SDQ in different waves of the LSIC [31]. For instance, the self-reported youth version of the SDQ was used in the LSIC wave 10 and wave 11 for participants aged 11–17 years. In this study, we used the SDQ prosocial subscale score to follow a strength-based approach. Based on the SDQ scoring guide and previous literature [31,34], the LSIC study team categorised child self-reported SDQ prosocial subscale score as average/high (score = 6–10), slightly low (score = 5), and low (score = 0–4) [31,35]. Note that for prosocial subscale scores, ‘average/high’ indicates that clinically significant problems are unlikely, scores such as ‘slightly low’ reflect some problems, and ‘low’ scores indicate substantial risks of clinically significant problems [31,35]. In this study, for analytical purposes, we used three categories—average/high, slightly low, and low—and gave them the notifications 2, 1, and 0.
Community-level factors	
Area of residence	The Australian Statistical Geography Standard (ASGS) classifies Remoteness Areas into five categories of relative remoteness across the country—Major Cities of Australia, Inner Regional Australia, Outer Regional Australia, Remote Australia, and Very Remote Australia [36]. From the responses, we created the binary variable ‘Area of Residence’—‘major cities’ were coded as ‘1’ and ‘inner regional’, ‘outer regional’, ‘remote’, and ‘very remote’ were combined as ‘regional/remote’ (coded as 0).
The Indigenous Relative Socioeconomic Outcomes (IRSEO) index	The IRSEO index is comprised of socioeconomic outcomes (i.e., employment, education, income, and housing) and is used to estimate the socioeconomic status of Indigenous Australians living in each Indigenous area in Australia. The lowest IRSEO index (Quintile 1, 0–20%) signifies the most disadvantaged, and the highest IRSEO index (Quintile 5, 80–100%) indicates the most advantaged at the Indigenous area-level [37]

^1^ We grouped the potential explanatory variables into individual and community level variables. We specifically sought to include variables previously shown or hypothesised to be associated with strong social and emotional well-being for Indigenous people. Variables were also coded to be strengths-based to examine each variable as a protective factor.

**Table 2:** Sample characteristics.

Variables	*n*	%
Individual-level factors		
Age ^1^ Mean (SD)	14.04 (0.45)	
Gender		
Girls	178	48.8
Boys	187	51.2
Schooling		
Not in school	14	3.8
Private/Catholic	67	18.4
Public	284	77.8
Family cohesion		
Poor	70	19.2
Good	295	80.8
Indigenous identity		
Not so important	111	30.4
Important	254	69.6
Making friends easily		
No	41	11.2
Yes	324	88.8
Self-efficacy		
Low	40	11.0
High	325	89.0
SDQ pro-social subscale scores		
Low	38	10.4
Slightly low	43	11.8
Average/High	284	77.8
Community-level factors		
Remoteness		
Regional/Remote	250	68.5
Urban	115	31.5
IRSEO index ^2^		
Q1—Most disadvantaged	25	6.9
Q2	46	12.6
Q3	141	38.6
Q4	99	27.1
Q5—Most advantaged	54	14.8

^1^ Continuous variable. ^2^ The Indigenous Relative Socioeconomic Outcomes (IRSEO) index; the lowest index (quintile 1, 0–20%) signifies the most disadvantaged, and the highest index (quintile 5, 80–100%) indicates the most advantaged at the Indigenous area-level.

**Table 3:** Factors associated with self-harm (bivariate analysis).

	Yes (*n* (%))	No (*n* (%))	*p*-Value
**Individual-level factors**			
Age (mean (SD))	14.03 (0.03)	14.05 (0.02)	0.049
Gender			0.005
Girls	22 (73.3)	156 (46.6)	
Boys	8 (26.7)	179 (53.4)	
Schooling			0.963
Not in school	1 (3.3)	13 (3.9)	
Private/Catholic	6 (20.0)	61 (18.2)	
Public	23 (76.7)	261 (77.9)	
Family cohesion			<0.001
Poor	14 (46.7)	56 (16.7)	
Good	16 (53.3)	279 (83.3)	
Indigenous identity			0.642
Not so important	8 (26.7)	103 (30.8)	
Important	22 (73.3)	232 (69.2)	
Making friends easily			0.028
No	7 (23.3)	34 (10.2)	
Yes	23 (76.7)	301 (89.8)	
Self-efficacy			0.296
Low	5 (16.7)	35 (10.5)	
High	25 (83.3)	300 (89.5)	
SDQ pro-social subscale scores			0.044
Low	7 (23.3)	31 (9.3)	
Slightly low	3 (10.0)	40 (11.9)	
Average/High	20 (66.7)	264 (78.8)	
**Community-level factors**			
Area of residence			0.008
Regional/Remote	27 (90.0)	223 (66.6)	
Urban	3 (10.0)	112 (33.4)	
IRSEO quintile			0.212
Q1—Most disadvantaged	3 (10.0)	22 (6.6)	
Q2	4 (13.3)	42 (12.5)	
Q3	16 (53.3)	125 (37.3)	
Q4	6 (20.0)	93 (27.8)	
Q5—Most advantaged	1 (3.4)	53 (15.8)	

**Table 4:** Factors associated with suicidality (bivariate analysis).

	Yes (*n* (%))	No (*n* (%))	*p*-Value
**Individual-level factors**			
Age (Mean (SD))	13.86 (0.09)	14.05 (0.02)	0.266
Gender			0.050
Girls	11 (73.3)	167 (47.7)	
Boys	4 (26.7)	183 (52.3)	
Schooling			0.086
Not in school	2 (13.3)	12 (3.4)	
Private/Catholic	4 (26.7)	63 (18.0)	
Public	9 (60.0)	275 (78.6)	
Family cohesion			0.001
Poor	8 (53.3)	62 (17.7)	
Strong	7 (46.7)	288 (82.3)	
Indigenous identity			0.371
Not so important	3 (20.0)	108 (30.9)	
Important	12 (80.0)	242 (69.1)	
Making friends easily			0.006
No	5 (33.3)	36 (10.3)	
Yes	10 (66.7)	314 (89.7)	
Self-efficacy			0.252
Low	3 (20.0)	37 (10.6)	
High	12 (80.0)	313 (89.4)	
SDQ pro-social subscale scores			0.699
Low	1 (6.7)	37 (10.6)	
Slightly low	1 (6.7)	42 (12.0)	
Average/High	13 (86.7)	271 (77.4)	
**Community-level factors**			
Area of residence			0.048
Regional/Remote	11 (73.3)	239 (68.3)	
Urban	4 (26.7)	111 (31.7)	
IRSEO quintile			0.476
Q1—Most disadvantaged	2 (13.3)	23 (6.6)	
Q2	2 (13.3)	44 (12.6)	
Q3	6 (40.0)	135 (38.6)	
Q4	5 (33.3)	94 (26.9)	
Q5—Most advantaged	0 (0.0)	54 (15.4)	

**Table 5:** Protective factors against self-harm.

	Model 1 ^a^ OR (95% CI)	Model 2 ^b^ OR (95% CI)	Model 3 ^c^ OR (95% CI)	Model 4 ^d^ OR (95% CI)
**Individual-level factors**				
Age		0.84 (0.28, 2.50)		0.83 (0.27, 2.62)
Gender				
Girls		Ref.		Ref.
Boys		4.23 ** (1.57, 11.42)		3.95 ** (1.44, 10.81)
Family cohesion				
Poor		Ref.		Ref.
Strong		4.28 ** (1.68, 10.88)		4.41 ** (1.69, 11.44)
Making friends easily				
No		Ref.		Ref.
Yes		1.66 (0.50, 5.55)		1.94 (0.54, 6.88)
Self-efficacy				
Low		Ref.		Ref.
High		0.96 (0.25, 3.68)		0.90 (0.22, 3.56)
SDQ pro-social subscale scores				
Low		Ref.		Ref.
Slightly low		3.33 (0.58, 18.95)		3.42 (0.57, 20.36)
Average/High		3.81 * (1.01, 13.16)		3.30 * (1.03, 11.78)
**Community-level factors**				
Area of residence				
Regional/Remote			Ref.	Ref.
Urban			10.05 * (1.49, 67.7)	6.97 * (1.34, 36.10)
Measure of variation				
Variance (SE)	2.60 (0.535)	1.35 (0.195)	2.15 (0.182)	1.29 (0.214)
ICC (%)	38.4	29.1	43.6	28.2
PCV (%)	Ref.	48.1	17.3	26.9
MOR	4.62	3.01	4.02	2.94
Model fit statistics				
AIC	206.52	195.24	199.13	189.17

^a^ Model 1 (the empty model) was fitted without explanatory variables. ^b^ Model 2 was adjusted for individual-level variables only. ^c^ Model 3 was adjusted for community-level variables only. ^d^ Model 4 was adjusted for both individual- and community-level variables. * *p* < 0.05, ** *p* < 0.01, *** *p* < 0.001. SE—standard error, ICC—intraclass correlation coefficient, PCV—proportional change in variance, MOR—median odds ratio.

**Table 6:** Protective factors against suicidality.

	Model 1 ^a^ OR (95% CI)	Model 2 ^b^ OR (95% CI)	Model 3 ^c^ OR (95% CI)	Model 4 ^d^ OR (95% CI)
**Individual-level factors**				
Age		4.27 (0.70, 25.99)		4.29 (0.70, 26.21)
Gender				
Girls		Ref.		Ref.
Boys		5.87 * (1.14, 30.14)		5.71 * (1.09, 29.91)
Family cohesion				
Poor		Ref.		Ref.
Strong		4.63 * (1.09, 19.57)		4.57 * (1.07, 19.40)
Making friends easily				
No		Ref.		Ref.
Yes		2.07 (0.38, 11.21)		2.10 (0.38, 11.47)
**Community-level factors**				
Area of residence				
Regional/Remote			Ref.	Ref.
Urban			1.97 (0.35, 11.15)	1.18 (0.21, 6.82)
Measure of variation				
Variance (SE)	2.61 (0.227)	1.16 (0.252)	1.99 (0.229)	1.14 (0.251)
ICC (%)	44.2	26.1	47.6	25.8
PCV (%)	Ref.	55.6	23.8	56.3
MOR	4.64	2.78	3.81	2.75
Model fit statistics				
AIC	125.05	120.83	118.29	122.15

^a^ Model 1 (the empty model) was fitted without explanatory variables. ^b^ Model 2 was adjusted for individual-level variables only. ^c^ Model 3 was adjusted for community-level variables only. ^d^ Model 4 was adjusted for both individual- and community-level variables. * *p* < 0.05, ** *p* < 0.01, *** *p* < 0.001. SE—standard error, ICC—intraclass correlation coefficient, PCV—proportional change variance, MOR—median odds ratio.

## Data Availability

Restrictions apply to the availability of these data. Data was obtained from the National Centre for Longitudinal Data (NCLD) and Australian Data Archive (ADA) Dataverse, and are available at https://dataverse.ada.edu.au/dataverse/lsic (accessed on 25 April 2022) with the permission of NCLD and ADA.
